# Palliative long-term abdominal drains vs. large volume paracentesis for refractory ascites secondary to cirrhosis: protocol for a definitive randomised controlled trial (REDUCe2 study)

**DOI:** 10.1186/s13063-025-08873-z

**Published:** 2025-06-04

**Authors:** Yazan Haddadin, Vasso Anagnostopoulou, Stephen Bremner, Helena Harder, Rachel Starkings, Debbie Lambert, Alison Porges, Nicky Perry, Wendy Wood, Amy Arbon, Heather Gage, Matthew Glover, Lucia Macken, Malcolm Johnston, Bhaskar Ganai, Dhiraj Joshi, Ben Hudson, Claire Butler, Alison Richardson, Mark Wright, Wendy Prentice, Alastair O’Brien, Joan Bedlington, Shani Steer, Tom Gaskin, Sumita Verma

**Affiliations:** 1 Department of Clinical and Experimental Medicine, Brighton & Sussex Medical School, University of Brighton and University of Sussex, Brighton, BN1 9PX UK; 2 Department of Gastroenterology and Hepatology (Digestive Diseases), University Hospitals Sussex NHS Foundation Trust, Royal Sussex County Hospital, Brighton, BN2 5BE UK; 3 Department of Primary Care and Public Health, Brighton & Sussex Medical School, Brighton, BN1 9PX UK; 4 Sussex Health Outcomes Research and Education in Cancer (SHORE-C), Brighton & Sussex Medical School, Brighton, BN1 9PX UK; 5 Brighton and Sussex Clinical Trials Unit, Department of Clinical and Experimental Medicine, Brighton & Sussex Medical School, Brighton, BN1 9PX UK; 6 Surrey Health Economics Centre, University of Surrey, Guildford, GU2 7XH UK; 7 Department of Radiology, University Hospitals Sussex NHS Foundation Trust, Royal Sussex County Hospital, Brighton, BN2 5BE UK; 8 The Exeter Liver Centre, Royal Devon and Exeter NHS Foundation Trust, Devon, EX2 5DW UK; 9 Centre for Health Services Studies, University of Kent, Canterbury, CT2 7 NZ UK; 10 School of Health Sciences, University of Southampton, Southampton, SO17 1BJ UK; 11 University Hospital Southampton, Southampton, SO16 6YD UK; 12 Department of Palliative Care, Kings College Hospitals NHS Foundation Trust, London, SE5 9RS UK; 13 Institute of Liver and Digestive Health, University College London, London, W1G 8EA UK; 14 PPI, LIVErNORTH, Durham, DH9 0SN UK; 15 PPI Member, London, UK

**Keywords:** Ascites, End-stage liver disease, Paracentesis, Palliative care, Quality of life, Caregivers, Patient reported outcome measures, Cost-effectiveness analysis

## Abstract

**Background:**

Ascites remains the most common complication of cirrhosis and a frequent reason for hospitalisation in advanced chronic liver disease (ACLD). Ascites is associated with significant symptom burden, caregiver workload and poor health-related quality of life (HRQoL). Once refractory to treatment, median survival is poor. Many with refractory ascites (RA) will neither receive a transjugular intrahepatic portosystemic shunt (TIPS) nor a liver transplant. Palliative care remains underutilised and evidence-based interventions focused on improving HRQoL are clearly needed. The standard of care for RA is repeated hospital ascites drainage with large volume paracentesis (LVP). Our earlier feasibility randomised controlled trial (RCT) (REDUCe) showed acceptability of palliative tunnelled long-term abdominal drains (LTADs), as well as preliminary evidence of safety and efficacy. The current REDUCe2 trial is a definitive national study designed to assess the impact of palliative LTADs on HRQoL in patients with RA due to ACLD.

**Methods/design:**

The REDUCe2 study is a pragmatic, multicentre, open-label, mixed-methods, superiority RCT being conducted in England, Scotland and Wales. Patients with RA secondary to ACLD who are ineligible for a liver transplant or TIPS will be randomised 1:1 to receive a LTAD or continue the current standard of care (LVP). Fortnightly home research visits will be conducted for 12 weeks in both arms. The primary outcome will be liver specific HRQoL assessed at 12 weeks using the Short Form Liver Disease Quality of Life questionnaire (SFLDQoL). Secondary outcomes include assessment of symptom burden (Ascites Questionnaire), health utilities (EQ-5D-5L tool), caregiver workload (Caregiver Roles and Responsibilities Scale—CRRS questionnaire), safety (including infection, acute kidney injury and other clinical outcomes), health resource utilisation and acceptability of the intervention by patients, caregivers and healthcare professionals. We aim to recruit a total of 310 patients (155 in each arm).

**Discussion:**

Effective palliative care provision remains an unmet need in ACLD. The REDUCe2 study, the largest palliative interventional trial in the UK, aims to address this inequity for this vulnerable and underserved cohort. It has the potential to generate high quality evidence to optimise and enhance palliative care in RA.

**Trial registration:**

ISRCTN26993825, date registered: 15/08/2022.

**Supplementary Information:**

The online version contains supplementary material available at 10.1186/s13063-025-08873-z.

## Background 

Liver-related deaths in England have increased more than three-fold since 1971 [1], with hospital admissions due to liver disease rising by 51.5% in the financial year ending in 2023 compared to the previous decade [2]. Ascites remains the most common complication of advanced chronic liver disease (ACLD), and the main driver for hospitalisation [3, 4]. Refractory ascites (RA) is defined as diuretic-resistant ascites due to lack of response to optimal medical management, or diuretic-intractable ascites due to medication-induced side effects [5]. Up to one-third of patients with ascites will develop RA.[6, 7] Transplant free survival in RA remains poor [6–9]. Unfortunately, many with RA will neither receive a TIPS nor a liver transplant [7, 8, 10, 11].

Ascites is one of the main drivers of impaired health-related quality of life (HRQoL) in patients with ACLD [12–14]. Despite the poor prognosis associated with RA and the high symptom burden, only a minority of patients with ACLD receive palliative care, often only in the last days or weeks of life [8, 15, 16]. Poorer HRQoL in patients with cirrhosis and ascites independently predicts both the 12-month mortality as well as unplanned hospitalisation [9, 17]. In addition to the impact on patients’ well-being, ACLD has an impact on caregivers, who have a higher care burden, lower quality of life and higher incidence of anxiety and depression, compared with the general population [13, 18].

The current standard of care (SOC) for RA is repeated hospital attendance every few weeks for ascites drainage through a temporary drain (large volume paracentesis LVP) [19, 20]. Our Patient and Public Involvement (PPI) group describe feeling stigmatised in hospital, and LVPs as being “unbearably painful,” “devastating” and “traumatic.” Repeated hospitalisations could be particularly distressing if patients are confronting a life-limiting diagnosis. The SOC is also financially costly, a UK study of 45,000 individuals dying from cirrhosis, revealed that one-third required an LVP or more in their last year of life, costing the National Health Service (NHS) over £21,000 per person [21].

Palliative long-term abdominal drains (LTADs) are standard of care in malignant ascites [22–25]. These tunneled drains allow community nurses or caregivers to drain small volumes of ascitic fluid at home. Compared to LVP, LTADs have the potential advantage of improving HRQoL by reducing ascites-related-hospitalisation and improving symptom control and could also be cost effective [22–25]. Their use in ACLD, however, has been limited due to the scarcity of evidence, largely due to a perceived infection risk, especially peritonitis, and uncertainty regarding community management [26]. However, a recent systematic review [27], case series [28], as well as an earlier feasibility trial [29], have provided preliminary evidence of acceptability, safety, efficacy, and cost-effectiveness of LTADs in ACLD. Currently, pending definitive evidence, the National Institute for Health and Care Excellence (NICE) recommends the use of LTADs in ACLD only in the context of special arrangements (i.e. in research or closely audited practice) [30].

Delivering palliative interventional trials in ACLD can be challenging [31]. However, the lack of evidence-based interventions is a factor in the inequity of palliative care provision for patients with ACLD, hence the need for this trial.

## Methods/design

### Primary objective

The primary objective is to assess whether palliative LTADs result in better disease-specific HRQoL compared to LVP in patients with RA due to ACLD.

### Secondary objectives

Secondary objectives are to assess the impact of LTADs vs LVP on.

- Incidence of infection (especially peritonitis)
- Symptom burden
- Caregiver workload
- Health resource utilisation
- Generic HRQoL
- Health utilities and cost per quality-adjusted life year (QALY)
- Perceptions of LTADs and LVP by patients, informal caregivers and healthcare professionals (HCPs) through qualitative interviews.

### Study design and setting

The REDUCe2 study is a mixed-methods, multicentre, open-label, superiority RCT being conducted in three United Kingdom (UK) nations (England, Wales and Scotland). Recruitment will take place over approximately four years with a three-month follow-up period for each participant. The study aims to recruit across 35 UK NHS Trusts (secondary/tertiary centres and district general hospitals), with a target sample size of 310 patients. Study sites participating in the trial at the time of manuscript submission are listed in Appendix 1. Follow-up research visits will take place in community settings (when possible) and will require close liaison between hospital-based research teams and local community partners.

### Characteristics of participants

**Fig. 1:**
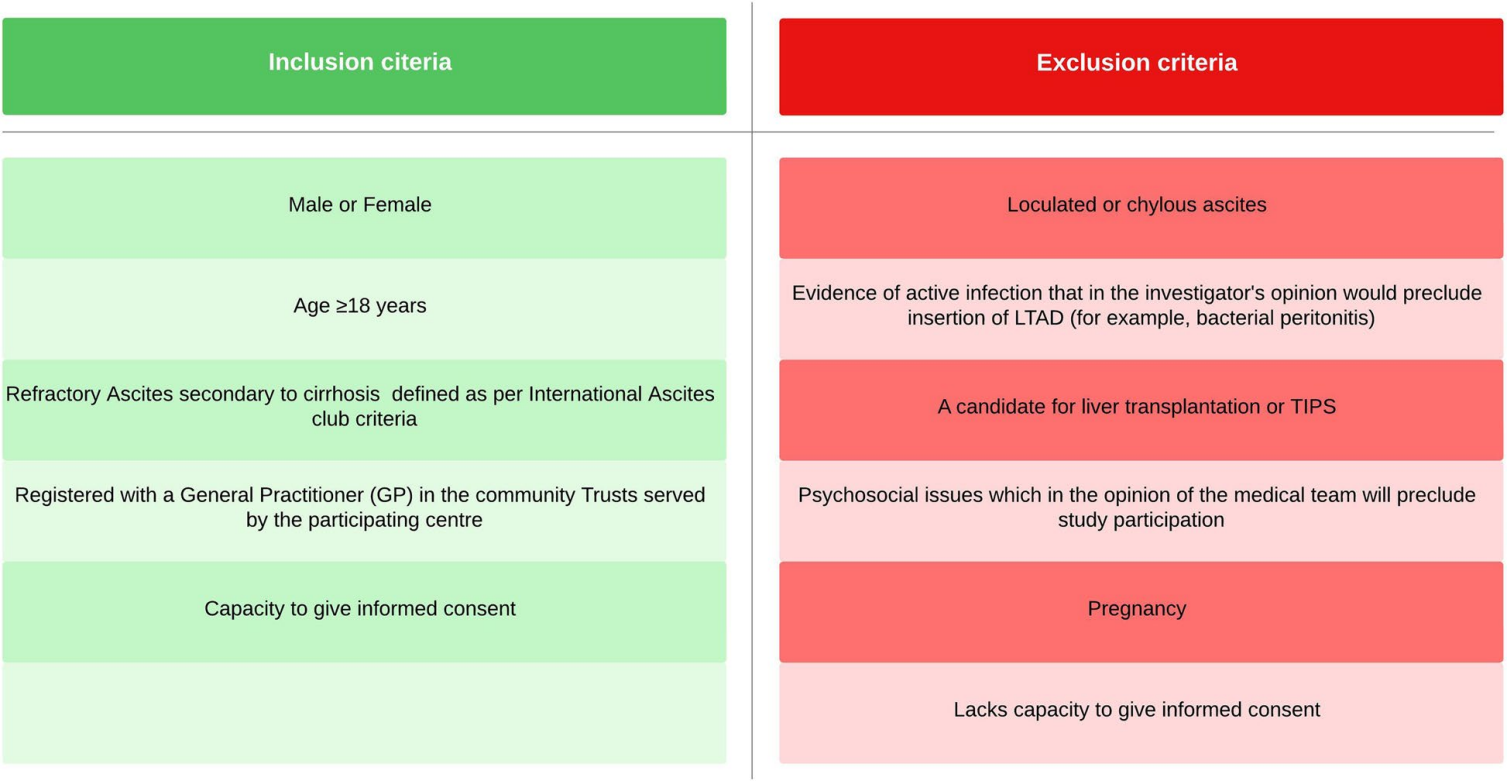
Inclusion and exclusion criteria. Legend: Abbreviations: LTAD long-term abdominal drain, TIPS transjugular intrahepatic portosystemic shunt

The study participants will include patients with RA due to ACLD who are not currently candidates for liver transplantation or TIPS. Study inclusion and exclusion criteria are shown in Fig. 1. Non-English-speaking patients and those residing in long-term care facilities are also eligible. Participants in other trials can be recruited, provided there is no conflict between study protocols and the participant is not overburdened. Informal caregivers will be encouraged to take part in the study, but their absence will not preclude recruitment.

### Participant timeline

**Fig. 2:**
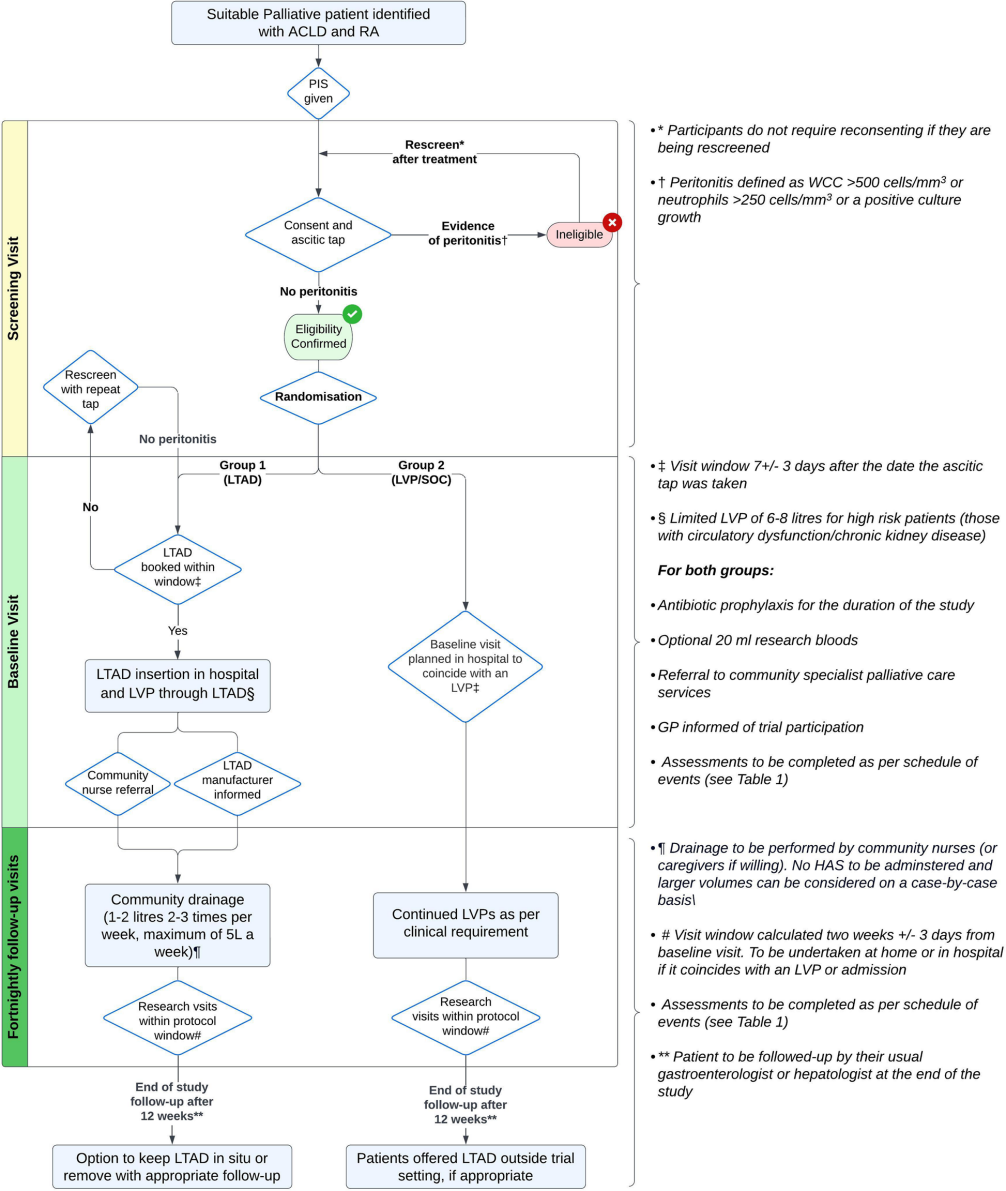
Participant flowchart. Abbreviations: ACLD advanced chronic liver disease, RA refractory ascites, PIS participant information sheet, LTAD long-term abdominal drain, LVP large volume paracentesis, SOC standard of care

The study timeline is shown in Fig. 2 along with a schedule of events detailed in Table 1.

**Table 1:**
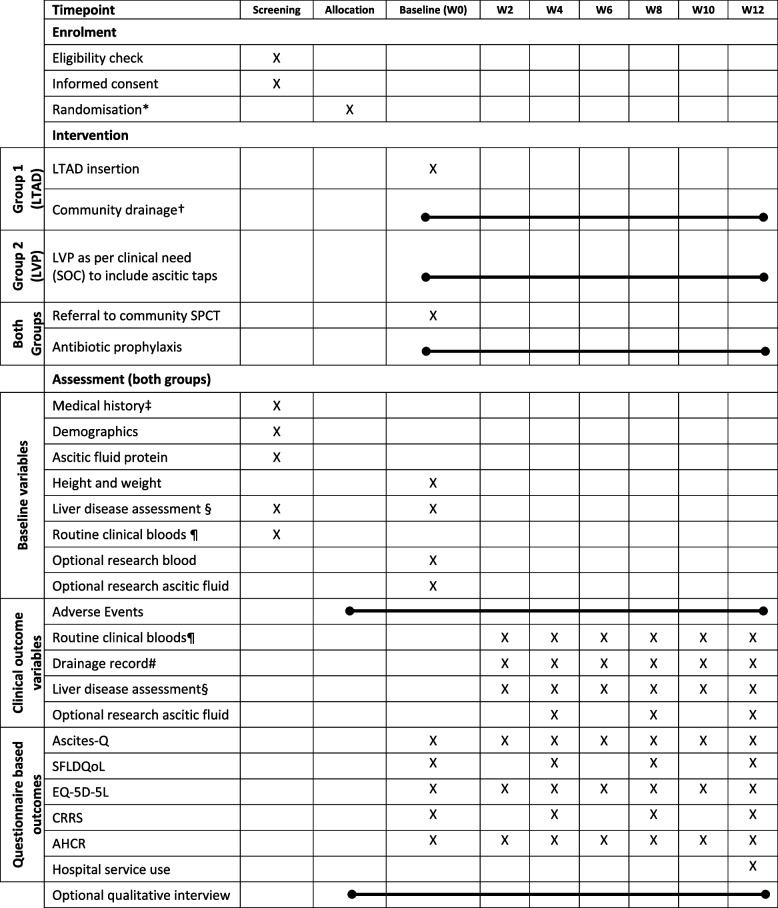
Schedule of events

Timepoints: visit windows calculated from baseline (+/-3 days)

* Randomisation once ascitic culture rules out peritonitis and eligibility confirmed.

† As per protocol – initially to drain 1-2 litres, two to three times a week, maximum of 5L a week

‡ To include reasons for TIPS/transplant ineligibility and assessment of alcohol and substance use

§ To include liver prognostic scores (Child Pugh score, MELD and UKELD) and liver complications such hepatocellular carcinoma, hepatic encephalopathy and variceal bleeding

¶ To include full blood count, urea and electrolytes, liver function tests, C reactive protein and INR. Haemostatic function to be corrected prior to LTAD insertion as per protocol

#Drainage record in LTAD group based on drain diaries, LVP group based on hospital records

*Abbreviations* **–** *LTAD* Long term abdominal drain, *LVP* Large volume paracentesis, *SFLDQoL* Short Form Liver Disease Quality of Life, *CRRS* Caregiver Roles and Responsibilities Scale, *AHCR* Ambulatory and Home Care Record

### Screening visit and consent

#### Screening visit

Prospective eligible patients will be identified through various pathways, including LVP day-case units, outpatient clinics, inpatients, multidisciplinary team (MDT) meetings, and palliative care referrals. A participant information sheet (PIS) will be provided to potential participants, and adequate time given for them to read and ask questions. If willing to participate, they will be invited to attend a hospital appointment and written informed consent will be received. For patients unable to provide written consent, verbal consent will be witnessed and countersigned by a third party according to Good Clinical Practice (GCP). Medical history, baseline data (including reasons for transplant/TIPS ineligibility) will be recorded and a diagnostic ascitic tap performed to exclude peritonitis.

Capacity regarding continuation in the study will be assessed at each follow-up visit. For those in England and Wales, verbal consent will be sought, and in case capacity is lost, a pre-agreed consultee (usually a caregiver) can be approached to ensure whether ongoing participation is in the patient’s best interest. For those in Scotland, the initial consent will be valid for the duration of the study, in accordance with Scottish law.

#### Caregiver consent

If available, and with the patient’s consent, informal caregivers will be approached and invited to participate in the trial. They will be given a caregiver PIS, after which written consent will be received for the questionnaire-based assessments and optional qualitative interview. Copies of the consent forms are included in Appendix 2.

### Randomisation

Once an ascitic tap has excluded peritonitis (defined as ascitic white cell count > 500 cells/mm^3^ or a neutrophil count > 250 cells/mm^3^ or a positive ascitic-fluid culture result)[19] participants will be registered by the local teams on an electronic data capture platform (Ennov:MACRO™) [32] and then randomised to receive either LTAD or standard of care (LVP) in a 1:1 ratio using Sealed Envelope™ [33]. The computer-generated software will confirm the patient’s treatment group allocation. The allocation will be minimised (i.e. dynamically generated) on sex and Child–Pugh Score (CPS) with an 80% probability of assigning participants to the group that balances these factors out. This will ensure comparability of the two groups. Only the trial manager, Chief Investigator (CI), and central research team will be aware of the exact sequence of the most recent allocations. Blinding researchers or participants is not feasible due to the nature of the intervention. However, the senior trial statistician will remain blinded until final data analysis, with adverse events (AEs) coded to prevent unintentional unblinding.

### Group 1 (LTAD)

Two routinely available LTADs will be used in the study. Rocket Medical plc (Watford, UK) Indwelling Peritoneal Catheters (IPCs)[34] and Becton Dickinson (New Jersey, USA) PeritX™ drains [35]. Choice of LTAD will be independently determined by each site’s research team.

#### LTAD insertion (baseline visit)

LTAD insertion must occur within 10 days of the ascitic tap. If this is not possible then a rescreening visit will be necessary to repeat the ascitic fluid analysis. Baseline assessments can be completed on the day of LTAD insertion. The procedure for LTAD insertion has been previously described [36], and will be performed by an interventional radiologist or an appropriately trained clinician. This is expected to be a day case procedure at most sites. Haemostatic parameters will be corrected according to local NHS trust policy prior to insertion. At sites where this is unavailable, patients will be transfused two units of fresh frozen plasma (FFP) when the international normalized ratio (INR) is ≥ 1.5 and up to two units of platelets if the platelet count is ≤ 50 × 10^9^/mm^3^.

#### Post LTAD insertion care

After insertion, participants will undergo ascitic fluid drainage through the LTAD in hospital, with human albumin solution (HAS) cover as per their usual LVP protocol and volume. The aim will be to safely drain the ascitic fluid, minimise the risk of leakage and cellulitis post insertion, and facilitate community management of ascites. However, in high-risk patients (those with circulatory dysfunction or chronic kidney disease) total drainage volume should be limited to a maximum of six to eight litres.

**Fig. 3:**
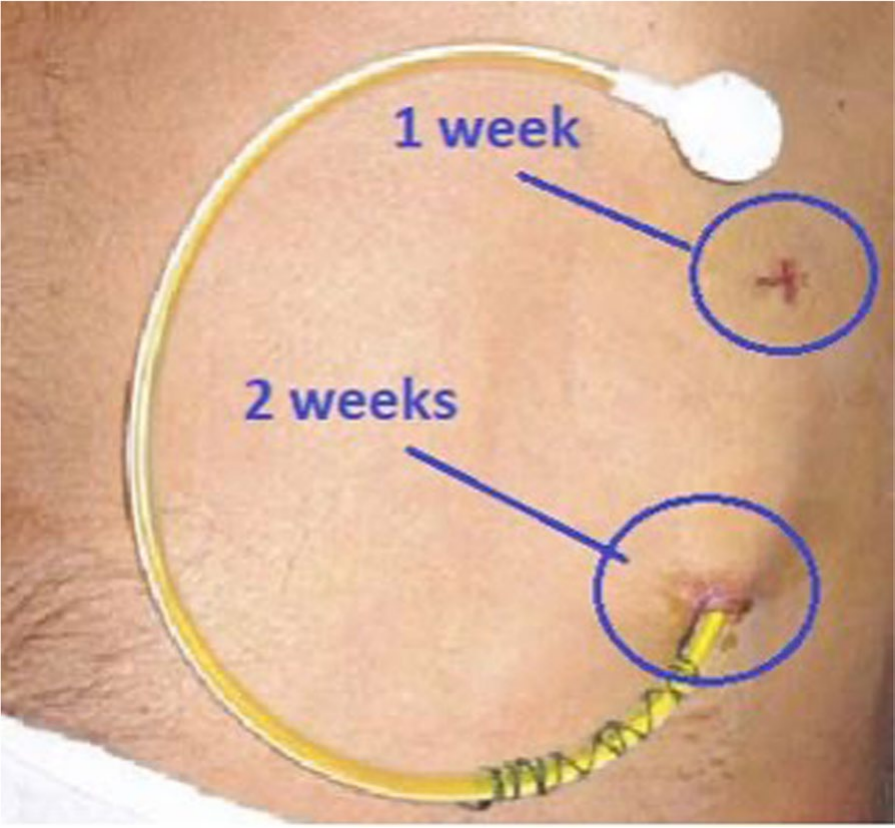
LTAD in situ with suggested timeline for suture removal

Effective communication with all relevant care teams is essential after LTAD placement. Participants and caregivers will receive clear information about the LTAD, including its use, suture removal timeline (Fig. 3), and the start of community visits. Contact details for local care teams will be provided, including details on how to access support in and out-of-hours.

#### Community LTAD management

Participants will receive an initial supply of compatible drainage kits and specific trial paperwork, such as drainage diaries for community nurses to record the volume and frequency of drainage. If keen and willing, caregivers will be given an opportunity to perform the drainage themselves, supported by the community nursing teams. However, the diaries should only be completed by the community nurses, who will perform risk assessments during their home visits and raise any concerns regarding the LTAD with the research team.

Frequency and volume of drainage through the LTAD in the community will be guided by the patient’s symptoms but generally should not exceed one to two litres at each visit and no more than two to three times a week (i.e. five litres per week). This was initially based on volumes used in malignant ascites [22], but was also sufficient for ascites management in 90% of patients in the earlier feasibility study [29]. In the minority needing drainage of larger volumes, an LVP via the LTAD can be arranged in hospital, including HAS administration. Subsequent drainage volumes can then be increased in the community on a case-by-case basis.

LTAD removal should only be considered at the participant’s request, or in the event of certain complications, for example intractable infection. Continued trial participation will be encouraged, despite LTAD removal. Re-insertion of LTADs can be organised if necessary, on a case-by-case basis. Appendix 3 summarises potential complications associated with the LTAD and a recommended management protocol for each.

### Group 2 (LVP)

The control arm, intermittent LVP, is the current accepted SOC for managing patients with RA [19], typically performed in a dedicated medical day unit. Depending on site policy, patients require referral from an HCP or could self-refer for the procedure. The patient’s baseline visit will be scheduled to take place within 10 days of screening to coincide with an LVP visit in hospital.

The frequency of LVPs will depend on clinical need. Trained HCPs are expected to perform LVPs following national and local guidelines, as previously described [19]. Routine measurement of haemostatic function is not recommended [19].

### Study follow-up (both groups)

A letter confirming research participation in the trial and group allocation will be sent to each patient’s general practitioner (GP).

### Antibiotic prophylaxis

Participants in both groups will receive antibiotic prophylaxis for the duration of the study to reduce infection risk and hospitalisation. The choice of antibiotic will be left to the attending healthcare team and depend on participant’s risk profile as well as local microbiology guidance.

### Referral to palliative care

All participants will be referred to community palliative care services as standard, without a requirement for current or immediate specialist palliative care needs.

### Fortnightly home research visits

Both groups will receive home visits at fortnightly intervals (± 3 days) conducted by GCP trained staff for safety monitoring and data collection (Table 1). Visits are planned in the community but can be carried out in hospital if these coincide with an admission or a planned LVP. During the visits the drainage diaries of LTAD patients will be collected. The visits will also provide researchers with an opportunity to ensure protocol adherence, as well as to troubleshoot and report any adverse events (AEs). Routine clinical bloods will be collected unless the patient declines or is too unwell, where it may be deemed inappropriate. No specific interventions would be prohibited as part of the protocol.

### Primary endpoint/outcome

The primary outcome is the difference in the overall mean of the disease-specific HRQoL scores between both groups assessed at 12 weeks using the Short Form Liver Disease Quality of Life (SFLDQoL) questionnaire [37].

### Secondary endpoints/outcomes

- Cumulative peritonitis incidence
- Symptoms assessed using the Ascites-Questionnaire (Ascites-Q)[38]
- Informal caregiver impact assessed using the Caregiver Roles and Responsibilities Scale (CRRS)[39]
- Health resource utilisation using the modified Ambulatory and Home Care Record (AHCR)[40] for community service use and patient record for hospital service use
- Generic HRQoL assessed using the 36-item Short Form Survey (SF-36) embedded in the SFLDQoL[37] and the EQ-5D-5L[41]
- Cost-utility analysis based on QALYs using EQ-5D-5L
- Patient, caregiver and HCP perceptions/perspectives of LTAD and LVP using qualitative methods
- Assessing predictors of mortality and infection using blood and genetic biomarkers

### Questionnaire-based assessments

These will be administered as per schedule of events (Table 1) during the fortnightly home research visits. This is largely consistent with our earlier feasibility study [29].

### Liver-specific HRQoL

This will be assessed at baseline and every four weeks using the SFLDQoL [37]. The SFLDQoL questionnaire is the only validated patient-reported outcome for ACLD and predicts mortality with accuracy comparable to MELD scores [9]. It includes 36 liver specific items across nine domains (distress, stigma, memory, symptoms and effects of liver disease, sleep, hopelessness, loneliness, and sexual function) scored on a 0–100 scale, where higher scores indicate better HRQoL. Due to low completion rates in the feasibility trial [29], sexual function data will be excluded from analysis but still recorded, should participants wish to answer. The questionnaire will be completed on paper or read out by researchers for patients who are too unwell. It takes 15–20 min to complete. The questionnaire also incorporates the 36-item Short Form Survey (SF-36) [42], which assesses generic HRQoL across eight domains, forming the two physical and mental component scores (PCS and MCS). The primary outcome only includes the liver-specific HRQoL measures and the PCS and MCS will be analysed separately. Caregivers or researchers can provide proxy scores when completing the questionnaire, if necessary. Although the primary outcome focuses on the SFLDQoL scores at three months, the questionnaire will be administered every four weeks to reduce the impact of missing data and improve the robustness of the statistical analysis model.

### Ascites-Q

Symptom burden will be assessed at baseline and fortnightly using the Ascites-Q tool validated in patients with ascites due to cirrhosis [38]. It is an 11-item questionnaire (abdominal pain, fullness, loss of appetite, satiety, nausea, shortness of breath, back pain, mobility, fatigue, sleeping issues, size of abdomen). Each symptom is assessed based on frequency (6-point Likert scale “never” to “always”) and discomfort (5-point Likert scale “not at all” to “a lot”). A higher summative score indicates a higher symptom burden. It takes 5–10 min to complete.

### Caregiver Roles and Responsibilities Scale (CRRS)

For caregivers who consent to participate in the study, the validated Caregiver Roles and Responsibilities Scale (CRRS) questionnaire will assess their care workload [39], completed at baseline and every four weeks. The CRRS, validated in the cancer setting, evaluates the impact on caregivers of providing support, across 41 items in five subscales: Support and Impact, Lifestyle, Emotional Health and Wellbeing, Self-care, and Financial Wellbeing, plus three standalone items. For employed caregivers, an additional Jobs and Career subscale can be calculated. The total CRRS score ranges from 0 to 152, with lower scores indicating higher caregiver burden. Missing data can be prorated if the completion rate is over 50% per subscale and 80% overall. It takes 10–15 min to complete.

#### EQ-5D-5L

EQ-5D-5L will also be administered at baseline and fortnightly visits. It has five dimensions (mobility, self-care, usual activities, pain/discomfort, and anxiety/depression) rated on a 5-point Likert scale (“no problems” to “severe problems/ unable”) [41]. Responses will be converted to a health utility value to calculate QALYs. The tool also includes a 20 cm vertical Visual Analogue Scale (VAS), capturing the patient’s overall perception of their health with range 0 (worst) to 100 (best). It takes 5 min to complete.

### Ambulatory and Home Care Record (AHCR)

Out-of-hospital service use will be collected using a customised version of the AHCR [40]. The AHCR asks for the number of contacts in and out of the home with professionals or services, and informal care (hours per day) delivered by family or friends. It takes about 10 min to complete.

### Hospital Service Use Questionnaire (HSUQ)

Hospital use related to liver treatment (including drain insertion, drainage and complications) will be extracted by research staff from participant hospital records at the end of the study using a bespoke inhouse designed proforma distinguishing outpatient, accident and emergency (A&E), day case and overnight stays.

### Genetic and molecular analysis

Optional research blood samples (collected at baseline) and ascitic fluid samples (collected at baseline and four-weekly) will investigate potential biomarkers predicting outcomes in ACLD, such as infection and mortality. Consent for this optional aspect will be received separately. These biomarkers will include, but not be limited to, bacterial DNA, microbiota analysis, lipopolysaccharide binding protein, tumour necrosis factor, interleukin 6 and genetic testing. Removed LTADs will also be stored for biofilm analysis.

### Qualitative interviews

The aims of the qualitative component of the study are to explore the lived experience and acceptability of LVP and LTADs to patients, caregivers and HCPs.

Semi structured interviews with 30 patients (15 per group), 20 caregivers (10 per group) and 20 HCPs will be undertaken. Sample sizes are informed by the principles of information power [43]. Interviews will take place at different study timepoints ensuring patients have had at least one round of the intervention.

Information about the optional interview is included within study information sheets. HCPs will receive an information sheet solely about the interviews. All participating individuals will provide a separate, audio-recorded verbal consent for the interview.

Interview topic guides have been collaboratively developed with the PPI and clinical research team members. Topic guides for patients and informal caregivers will mirror each other, using broad prompts to ask about HRQoL and the experience of LTAD/LVP and decision-making at joining the study. The topic guide for HCPs will focus on the decision-making process for LTAD/LVP, and perceived impact, practical implications and implementation of the intervention. Completed interviews will be reviewed across the study to amend topic guides as necessary, incorporating any elements which seem to reoccur.

The interviews will be conducted by phone or video link and take between 15 and 45 min. To reduce patient burden, breaks will be allowed, and participants reminded they can stop the interview at any time. Interviews will be digitally recorded, transcribed and analysed.

### Communication skills training

Communication-skills training interventions can positively impact researchers involved in clinical trials [44–46]. Four communication workshops, open to all researchers involved in the trial, will be delivered during the recruitment period of the REDUCe2 study and address difficulties when discussing recruitment and topics around end-of-life, palliative care and death.

### Sample size calculation

The minimal clinically important difference (MCID) is the mean change in HRQoL scores for patients reporting a minimal yet perceptible change in health between the baseline and follow-up assessments [47]. In the ongoing LiverPal Study (one of the largest palliative trial in the USA [48], an MCID of 9 points on the FACT-Hep questionnaire was used [49]; and in a study assessing LTADs in cirrhosis,[50] an MCID of 10 points was chosen (SF-36 questionnaire[42]). While these studies show the use of MCID to inform sample size in palliative trials, neither of the tools selected are validated in ACLD. As we will be using the SFLDQoL [37], a sensitive and validated ACLD questionnaire, and to ensure a robust sample size, we have selected a MCID of 8 points.

In the earlier REDUCe trial [29], the pooled baseline mean across SFLDQoL domains (excluding sexual function) was 56.4 (SD = 26.1). With 93 participants in each group for the analysis, we will have 90% power for 5% significance to detect an adjusted difference in mean SFLDQoL scores of 8 points between the LTAD and LVP groups at the end of 3 months (effect size 0.31). This effect size falls within Cohen’s recommended cut offs for small (0.20) to moderate (0.50) effect size [51]. We assume a correlation between baseline and the 3 follow-up measurements of 0.48 which is the lower bound of the 95% confidence interval for the correlation from the feasibility data (point estimate 0.77) [29]. With an expected 40% attrition [29], we will recruit 310 participants in total for the trial. The sample size was calculated using Stata (version 17.0).

Following on from lessons learnt during the earlier feasibility study [29], to improve study retention, the protocol allows for proxy-scores to be collected on behalf of patients who may be too unwell to complete the questionnaires themselves. Secondly, appointment of consultees who can be approached in case of loss of capacity during the trial follow-up period will allow patients to be retained even in case of disease progression. Finally, to prevent recruitment late in their disease trajectory, we are providing communication skills training for researchers to facilitate difficult palliative care discussions.

### Harms and safety monitoring

Common Terminology Criteria of Adverse Events (CTCAE) version 4.03 [52] will be used to report any untoward medical occurrence in a trial participant, and its classification will depend on severity, seriousness, and relatedness to the intervention. Given the advanced nature of the illness, a high incidence of adverse events (AEs), including serious adverse events (SAEs) leading to patient death, is anticipated. While all adverse events and reactions must be documented in the electronic clinical case record form (eCRF), only potential Serious Adverse Reactions (SARs) require expedited reporting to the Brighton and Sussex Clinical Trials Unit (BSCTU) within 24 h. Any potential SARs will be immediately reviewed by the CI for relatedness and expectedness.

The Research Ethics Committee (REC) will be notified of any Suspected Unexpected Serious Adverse Reactions (SUSARs) and any urgent safety measures will be reported immediately to the REC and the University of Sussex (UoS), who is sponsoring the study. A list of the expected SARs that could be related to the insertion and management of LTADs along with anticipated harms that will be assessed systematically is included in Appendix 4. The CI will have ultimate responsibility for any reported SARs and SUSARs and will sign off any reports following a discussion with the local site PI. To minimise the risk of bias, given the open-label nature of the study, rates of adverse events will also be compared between the two groups irrespective of the assessed relatedness to the intervention. Adverse events (AE, SAE, AR, SAR) will be assessed through both direct and open questions at follow-up visits, and by reviewing fortnightly safety bloods and clinical records of patients at the end of the study. Anticipated harms of clinical interest will be reported irrespective of prevalence or seriousness. Other harms will only be reported if they are serious or above 5% prevalence.

### Data collection and management

Paper questionnaires and worksheets will be used for data collection across study sites. They will serve as source documents and transcribed onto the electronic data capture system MACRO™ [32]. The paper forms will be stored at study sites in fire-proof secure locked cupboards as per GCP requirement. All paper forms for the SFLDQoL and CRRS questionnaires will be scanned and sent to a central team at Sussex Health Outcomes Research and Education in Cancer (SHORE-C) who will conduct quality checks to ensure complete and accurate entry into the electronic database. The team will print their own copies of these questionnaires and store them in a locked office in a security-controlled building. These back-up copies will be confidentially destroyed once the study data has been published.

Additional source document verification for other questionnaires and clinical data will be carried out by the trial manager during on-site monitoring visits. Essential data will be kept for a minimum of five years, and research data kept in secure storage for ten years. Clinical outcomes will be self-reported and verified by research teams against linked medical records, with fortnightly bloods analysed by local site labs and reviewed by PIs.

The study will receive Research and Development confirmation of capacity and capability from all participating NHS Trust sites and expected to adhere to GCP guidelines, the approved study protocol, and any study specific SOPs. Participant data will also be entered onto the electronic data capture system. Participant data will be pseudoanonymised with identification numbers issued by the system. The SFLDQoL and CRRS questionnaires will be managed on a separate database housed on a secure UoS server. Data management will perform data checks on a regular basis for quality assurance.

### Data analysis

#### Statistical analysis

**Fig. 4:**
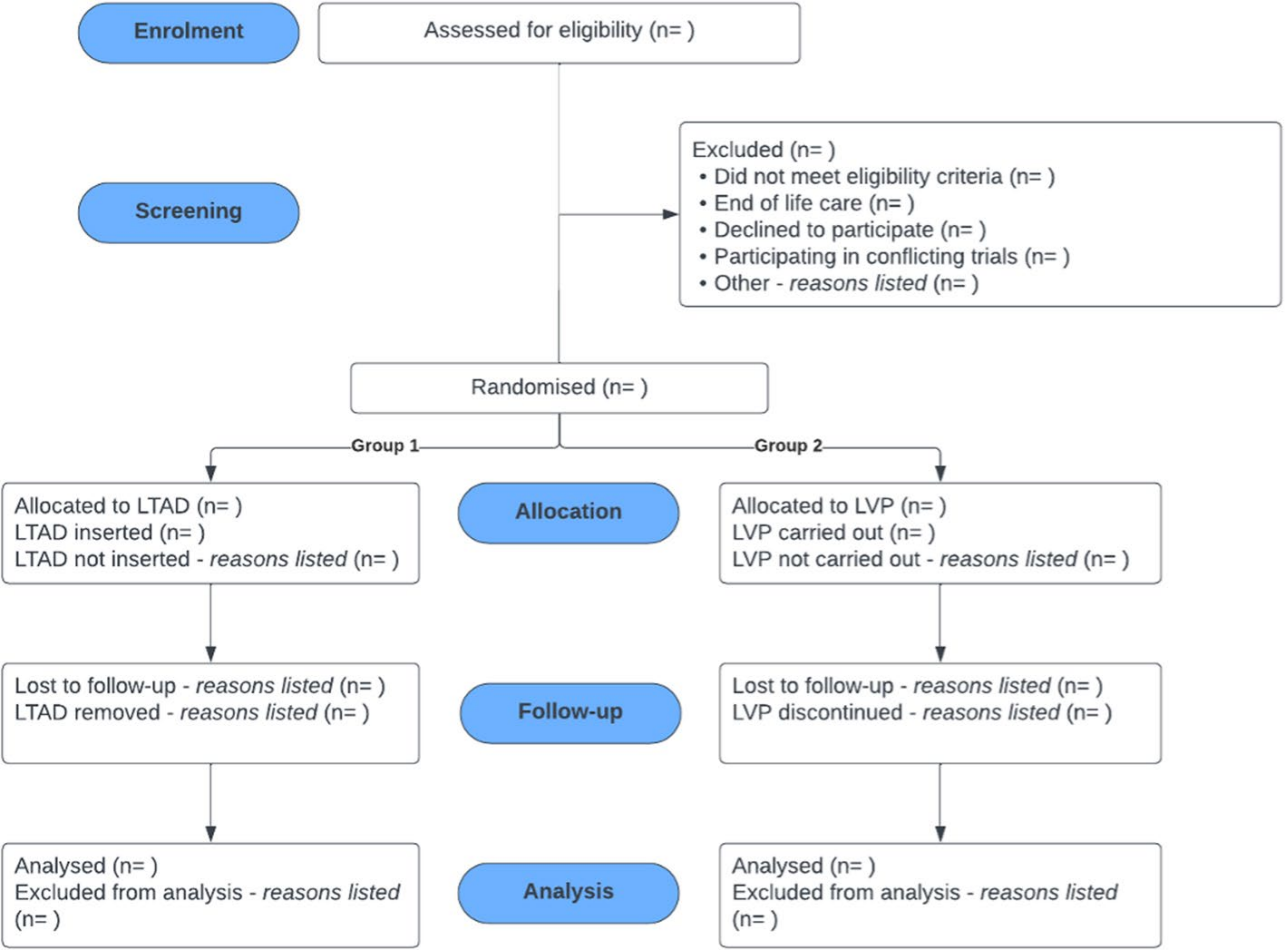
CONSORT diagram. Abbreviations: n number, LTAD long-term abdominal drain, LVP large volume paracentesis

The flow of patients through the trial will be depicted in a Consolidated Standards of Reporting Trials (CONSORT) diagram (Fig. 4). Descriptive statistics will be used to summarise the data using means and standard deviations for normally distributed data, medians and interquartile ranges for skewed continuous variables, and frequencies and percentages for categorical variables.

For the primary outcome, the analysis will follow intention-to-treat principles and will be conducted using a linear mixed effects model. All patients randomised through Sealed Envelope will be included in the group to which they were allocated regardless of the outcome or clinical trajectory.

This model will include fixed effects: time point, sex, Child Pugh score, randomisation group, and an interaction between randomisation group and time point, while adjusting for baseline SFLDQoL scores. A random effect for participant will be included to handle the correlation between repeated measurements across the study period. The model parameters will be estimated using restricted maximum likelihood, and treatment differences between groups at each follow-up will be reported along with 95% confidence intervals and p-values. Secondary outcomes will similarly be analysed using mixed effects regression models suited to the type of outcome.

Subgroup analyses will aim to stratify data based on patient and site-level characteristics. Patient-level factors will include liver prognostic scores baseline ascitic fluid protein level, socioeconomic status, and the presence of informal caregivers. Site-level data will include prior experience with LTADs, availability of dedicated LVP units, cirrhosis nurses, Palliative care MDTs for ACLD, and Improving Quality in Liver Services (IQILS) programme accreditation. Subgroup analyses will allow for the identification of potential effect modifiers or predictors of outcomes and changes in HRQoL.

Only in the case of significant protocol deviations, agreed with the CI, will participants be excluded from the final analysis. In accordance with MORECare guidance for palliative trials, attrition due to mortality, illness, and withdrawal will be detailed for each group [53]. Missing quantitative data will be assumed to be missing not at random (MNAR) unless evidence suggests otherwise. Imputation models will be utilised followed by sensitivity analyses using plausible adjustments to explore the potential impact of missing data and MNAR assumptions on treatment outcomes (multiple imputation with delta adjustment).

### Health economic analysis

The health economic analysis will adopt a health and social care perspective. Whole system costs for LTAD and LVP will be compared to assess if community drainage is resource saving compared to hospital drainage. Items of service use will be converted to costs (British pounds 2023) using nationally validated unit costs [54], and NHS reference costs [55]. Time spent by informal caregivers will be valued using replacement cost methods and applying the tariff for community support workers [56]. Costs for each main category of resource use (primary, community, hospital, social, voluntary, informal) will be reported as mean, standard deviation and median (range, IQR). Since patients will be in the study for different durations, the data will be standardised for fortnightly analysis, if necessary, for meaningful comparisons.

A within trial cost-effectiveness analysis will be conducted based on three-month follow-up. EQ-5D-5L utilities will be reported at each follow-up time point as mean and standard deviation. QALYs per patient will be calculated using the area under the curve approach. Use of QALYs in palliative care remains controversial, due to problems with conceptualising HRQoL, restrictions in life years gained and valuation of time. However, QALYs are widely used and in the absence of well recognised alternatives, they offer useful data for health policy makers [53, 57].

Differences in costs and QALYs will be estimated using linear mixed effects models, in line with the statistical approach to other outcomes (QALYs adjusted for baseline utility [58] and used to compute cost per QALYs of LTAD vs. LVP. Uncertainty will be characterised using probabilistic unit costs and non-parametric bootstrapping with replacement techniques in estimation processes.

### Qualitative data analysis

Thematic analysis supported by qualitative software (NVivo™) [59] will be used to develop overarching themes from the interviews. Interviews will be analysed separately for the three groups of participants (patients, informal caregivers and HCP) after which themes will be compared across participant groups to explore overlap or discrepancies. Utilising the process of triangulation [60], the findings of the qualitative arm will be used to contextualise the quantitative results as they relate to HRQoL.

### Ancillary and post-trial care

Routine clinical care of participants will not be affected by participation in the trial. At the end of the study, participants will continue routine care with their medical teams. Those randomised to LTAD can opt to retain it, or have it removed. Those on the SOC arm can be offered a LTAD outside the trial setting after study completion on a case-by-case basis. The sponsor will provide indemnity cover for negligent harm if applicable.

### Monitoring and trial committees

#### Site monitoring plan

The trial manager will conduct remote or in-person monitoring at each site, as outlined in a pre-approved Monitoring Plan. Monitoring frequency will depend on recruitment, withdrawals, and adverse events at each site. These visits will review participant enrolment, consent, eligibility and trial arm allocation, and ensure adherence to interventions, harm reporting, and data collection accuracy.

### Trial management group (TMG)

The TMG, consisting of research team members from each site, will be chaired by the CI and meet monthly. These meetings will troubleshoot any site issues including recruitment, review proposed changes to trial documents, and ensure timely trial completion.

### Trial Steering Committee (TSC)

The TSC, comprising independent members (two hepatologists one of whom will be the chair, a statistician, and PPI member), the trial CI, trial manager and trial statistician, will oversee trial conduct and recruitment on behalf of the sponsor and funder. Meeting biannually, the TSC will review study progression, considering safety reports issued by the Data Safety and Monitoring Committee (DSMC).

### Data Safety and Monitoring Committee (DSMC)

The DSMC, an independent committee including a hepatologist (chair), statistician, and palliative care physician, will review study data following its terms of reference. The DSMC will meet approximately every six months and address safety concerns, ethical issues, and adverse events, providing recommendations to the TSC.

### Role of PPI

The PPI group have been involved since the feasibility trial [29] and helped shape the research methodology, outcome measures and assessment tools. PPI members were co-applicants on the grant application, are part of the TMG and will be co-authors on any publication(s).

### Role of the sponsor and funder

The study sponsor and funder had no role in the study design, nor the collection, management, analysis and interpretation of data. They will not be involved in the writing of any reports. The sponsor however will ensure appropriate procedures are in place for reporting progress and safety monitoring and that there is a clear strategy for dissemination of research findings.

### SPIRIT guidelines

The SPIRIT reporting guidelines have been used in this manuscript [61], with the checklist found in Appendix 5 and the SPIRIT figure shown as Table 1.

## Discussion

Running a palliative RCT in ACLD is challenging, with barriers at all stages, including selection of appropriate outcome measures, recruitment, and addressing missing data. This may explain the lack of evidence-based palliative interventions in ACLD [31]. Encouragingly however, national and international guidelines, including NICE, have recently highlighted the need for further research on the use of palliative LTADs in RA [19, 30, 62]. The REDUCe2 study aims to address this knowledge gap and advance the field of palliative care in hepatology.

The trial has several strengths, one of which is its inclusive design to include district general, secondary and tertiary hospitals as well as participants from a range of socioeconomic backgrounds and sites with a high liver disease prevalence. This will enhance the study’s generalisability and validity. Another strength is the close collaboration with our PPI group in the trial design, which ensured selection of appropriate assessment tools and outcome measures, as well as incorporating caregivers’ perspectives. Finally, since this study cuts across healthcare boundaries, it will promote collaboration between hospital and community trusts, aligning with the vision of future healthcare delivery being closer to home [63].

The home research visits, however, are not without their challenges. Although critical for patient follow-up, especially in a palliative trial, they are logistically demanding. This has precluded trial participation for a few sites. However, direct delivery teams from some Clinical Research Networks have had capacity to assist sites with these visits. Additionally, each site is only expected to recruit a small number of patients per year and the study has a short follow-up period. Another contentious issue is that since patients with RA are a heterogeneous group, timely recruitment along their disease trajectory can be challenging. Rapid deterioration can occur, in keeping with the fluctuating nature of ACLD, and interventional procedures in the final days or weeks of patients’ lives may not be appropriate.

The potential impact of the successful completion of the REDUCe2 trial could be substantial. Besides providing high-quality evidence for the palliative management of RA in ACLD, it could also serve as a blueprint for future palliative trials in hepatology.

## Trial status

Current protocol v9.0 28.08.2024. Appendix 6 summarises the protocol amendments to date. The study commenced in May 2022 with a six-month study set up period, active recruitment starting in Nov 2022 and planned to run to Sept 2026 (Last Patient Last Visit), with a final 4 months for data analysis. An 18-month internal pilot was successfully completed in Jan 2024 (see Appendix 7 for STOP–GO criteria).

## Supplementary Information

Additional file 1. Appendices 1–7.

## Data Availability

In line with the 2018 Data Protection Act, any data collected as part of this trial will be kept strictly confidential. All study data will either be held on secure university and hospital computers or in a secure and locked location at the BSCTU. A final study report will be submitted to the REC Committee and NIHR for publication. Findings will be disseminated through local and international conferences and peer reviewed publication. Our results will likely inform national policy and NICE guidance. A lay summary of key findings, developed with PPI input, will be shared with study sites, patient organisations and participants. The study protocol and report will be available on the ISRCTN registry. Anonymised patient data and statistical code will be available from the sponsor or CI upon formal reasonable request.

## Abbreviations

A&E: Accident and emergency
ACLD: Advanced chronic liver disease
AE: Adverse event
AHCR: Ambulatory and home care record
Ascites-Q: Ascites Questionnaire
BSCTU: Brighton and Sussex Clinical Trials Unit
CI: Chief investigator
CPS: Child–Pugh Score
CRRS: Caregiver Roles and Responsibilities Scale
CTCAE: Common Terminology Criteria for Adverse Events
DSMC: Data Safety and Monitoring Committee
FFP: Fresh frozen plasma
GCP: Good clinical practice
GP: General practitioner
HAS: Human albumin solution
HCP: Healthcare professional
HRA: Health research authority
HRQoL: Health-related quality of life
HSUQ: Hospital Service Use Questionnaire
INR: International normalised ratio
IQILS: Improving Quality in Liver Services
ISRCTN: International Standard Randomised Controlled Trial Number
LTAD: Long-term abdominal drains
LVP: Large volume paracentesis
MDT: Multidisciplinary team
NHS: National Health Service
NICE: National Institute for Health and Care Excellence
NIHR: National Institute for Health and Care Research
PPI: Patient and public involvement
PI: Principal investigator
PIS: Participant Information Sheet
QALY: Quality-adjusted life year
RA: Refractory ascites
REC: Research Ethics Committee
RCT: Randomised controlled trial
SAE: Serious adverse event
SAR: Serious adverse reaction
SBP: Spontaneous bacterial peritonitis
SHORE-C: Sussex Health Outcomes Research and Education in Cancer
SFLDQoL: Short Form Liver Disease Quality of Life
SOC: Standard of care
SOP: Standard operating procedure
SUSAR: Suspected unexpected serious adverse reaction
TIPS: Transjugular intrahepatic portosystemic shunt
TMG: Trial management group
TSC: Trial Steering Committee
UoS: University of Sussex
UK: United Kingdom
VAS: Visual Analogue Scale
