# Correction: Palliative long-term abdominal drains vs. large volume paracentesis for refractory ascites secondary to cirrhosis: protocol for a definitive randomised controlled trial (REDUCe2 study)

**DOI:** 10.1186/s13063-025-08918-3

**Published:** 2025-08-06

**Authors:** Yazan Haddadin, Vasso Anagnostopoulou, Stephen Bremner, Helena Harder, Rachel Starkings, Debbie Lambert, Alison Porges, Nicky Perry, Wendy Wood, Amy Arbon, Heather Gage, Matthew Glover, Lucia Macken, Malcolm Johnston, Bhaskar Ganai, Dhiraj Joshi, Ben Hudson, Claire Butler, Alison Richardson, Mark Wright, Wendy Prentice, Alastair O’Brien, Joan Bedlington, Shani Steer, Tom Gaskin, Sumita Verma

**Affiliations:** 1 Department of Clinical and Experimental Medicine, Brighton & Sussex Medical School, University of Brighton and University of Sussex, Brighton, BN1 9PX UK; 2 Department of Gastroenterology and Hepatology (Digestive Diseases), University Hospitals Sussex NHS Foundation Trust, Royal Sussex County Hospital, Brighton, BN2 5BE UK; 3 Department of Primary Care and Public Health, Brighton & Sussex Medical School, Brighton, BN1 9PX UK; 4 Sussex Health Outcomes Research and Education in Cancer (SHORE-C), Brighton & Sussex Medical School, Brighton, BN1 9PX UK; 5 Brighton and Sussex Clinical Trials Unit, Department of Clinical and Experimental Medicine, Brighton & Sussex Medical School, Brighton, BN1 9PX UK; 6 Surrey Health Economics Centre, University of Surrey, Guildford, GU2 7XH UK; 7 Department of Radiology, University Hospitals Sussex NHS Foundation Trust, Royal Sussex County Hospital, Brighton, BN2 5BE UK; 8 The Exeter Liver Centre, Royal Devon and Exeter NHS Foundation Trust, Devon, EX2 5DW UK; 9 Centre for Health Services Studies, University of Kent, Canterbury, CT2 7NZ UK; 10 School of Health Sciences, University of Southampton, Southampton, SO17 1BJ UK; 11 University Hospital Southampton, Southampton, SO16 6YD UK; 12 Department of Palliative Care, Kings College Hospitals NHS Foundation Trust, London, SE5 9RS UK; 13 Institute of Liver and Digestive Health, University College London, London, W1G 8EA UK; 14 PPI, LIVErNORTH, Durham, DH9 0SN UK; 15 PPI Member, London, UK

**Correction: Trials 26, 193 (2025)**

**https://doi.org/10.1186/s13063-025–08873-z**

Following the publication of the original article [1], we were notified that only the first two lines of Table 1 were published.

Originally published Table 1:

**Table 1:**
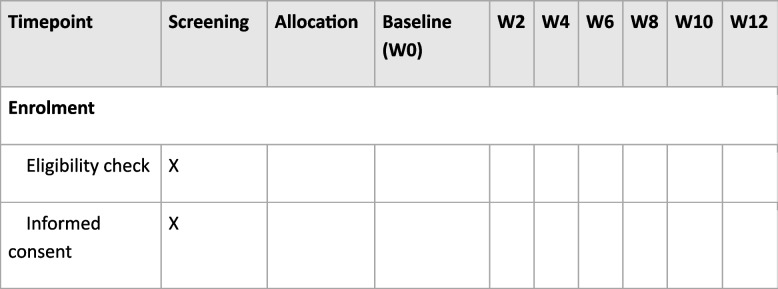
Schedule of events

Timepoints: visit windows calculated from baseline (± 3 days)

Corrected Table 1:

**Table 1:**
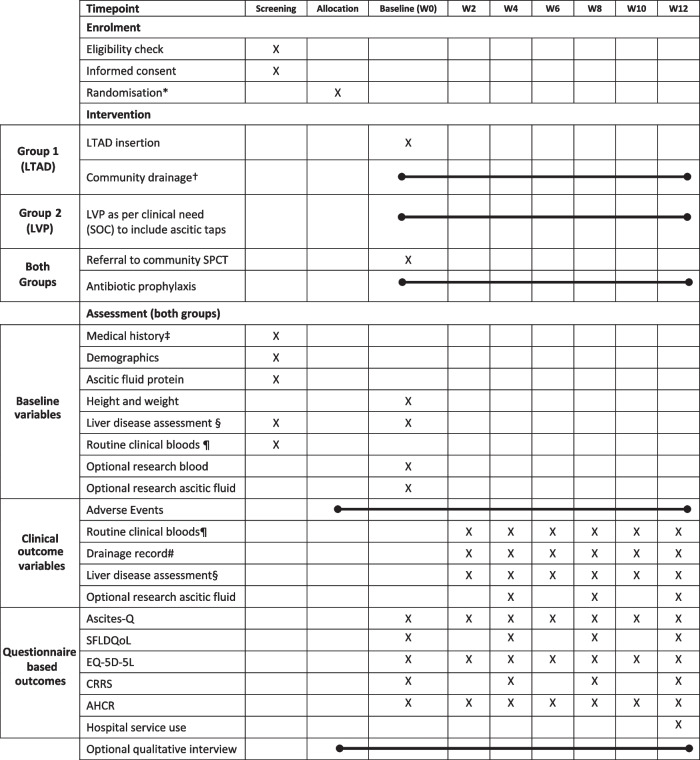
Schedule of events

**Timepoints: **visit windows calculated from baseline (+/−3 days)

*Randomisation once ascitic culture rules out peritonitis and eligibility confirmed

†As per protocol – initially to drain 1–2 litres, two to three times a week, maximum of 5L a week

‡To include reasons for TIPS/transplant ineligibility and assessment of alcohol and substance use

§To include liver prognostic scores (Child Pugh score, MELD and UKELD) and liver complications such hepatocellular carcinoma, hepatic encephalopathy and variceal bleeding

¶To include full blood count, urea and electrolytes, liver function tests, C reactive protein and INR. Haemostatic function to be corrected prior to LTAD insertion as per protocol

#Drainage record in LTAD group based on drain diaries, LVP group based on hospital records

**Abbreviations:** *LTAD* Long term abdominal drain, *LVP* Large volume paracentesis, *SFLDQoL* Short Form Liver Disease Quality of Life, *CRRS* Caregiver Roles and Responsibilities Scale, *AHCR* Ambulatory and Home Care Record

The original article was corrected.
