# Supplementary material for: Palliative long-term abdominal drains vs. large volume paracentesis for refractory ascites secondary to cirrhosis: protocol for a definitive randomised controlled trial (REDUCe2 study)
Source: Trials. 2025 Jun 4;26:193. doi: 10.1186/s13063-025-08873-z (PMC12139341; doi:10.1186/s13063-025-08873-z)
Supplement: Supplementary file 1 — Additional file 1. Appendices 1–7. [file 13063_2025_8873_MOESM1_ESM.zip › Appendix 1R1.docx]

**Appendix 1 –** Participating sites at the time of publication

| **Site Number** | **Trust** | **Hospital/Site** | **PI name** | **Date opened** |
| --- | --- | --- | --- | --- |
| **OPEN** | | | | |
| RSC01 | University Hospitals Sussex NHS Foundation Trust | Royal Sussex County Hospital | Prof Verma | 05/10/2022 |
| WOR19 | University Hospitals Sussex NHS Foundation Trust | Worthing Hospital | Dr Sam Thomson | 18/11/2022 |
| BOL21 | Bolton NHS Foundation Trust | Bolton Royal infirmary | Dr Mahesh Bhalme | 07/12/2022 |
| HUL04 | Hull University Teaching Hospitals NHS Trust | Hull Royal Infirmary | Dr Lynsey Corless | 19/12/2022 |
| QEG07 | NHS Greater Glasgow and Clyde Health Board | Queen Elizabeth Hospital Glasgow | Dr Rachael Swann | 10/01/2023 |
| UHS02 | University Hospital Southampton NHS Foundation Trust | Southampton General Hospital | Dr Mark Wright | 18/01/2023 |
| KCH18 | Kings College Hospitals NHS Foundation Trust | Kings College Hospital | Dr Vishal Patel | 30/01/2023 |
| HAI08 | NHS Lanarkshire | University Hospital Hairmyres | Dr Natasha McDonald | 08/02/2023 |
| ROY13 | Royal Devon University Healthcare NHS Foundation Trust | Royal Devon and Exeter Hospital | Dr Ben Hudson | 15/02/2023 |
| STG17 | St George’s University Hospitals NHS Foundation Trust | St George’s Hospital | Dr Arj Singanayagam | 09/03/2023 |
| PLY28 | University Hospitals Plymouth NHS Trust | Derriford Hospital | Dr David Sheridan | 20/03/2023 |
| ARI05 | NHS Grampian | Aberdeen Royal Infirmary | Ashis Mukhopadhya | 23/03/2023 |
| CUH25 | Cambridge University Hospitals NHS Foundation Trust | Addenbrookes | Dr Navjyot Hansi | 15/05/2023 |
| GST11 | Guy's and St Thomas' NHS Foundation Trust | St Thomas's Hospital | Dr Sreelakshmi Kotha | 25/05/2023 |
| GLO29 | Gloucestershire Hospitals NHS Trust | Gloucestershire Royal Hospital | Dr Duncan Napier | 30/05/2023 |
| RGH27 | Cwm Taf Morgannwg University Health Board | Royal Glamorgan Hospital | Dr Dai Samuel | 14/06/2023 |
| FRE26 | The Newcastle Upon Tyne Hospitals NHS Foundation Trust | Freeman Hospital | Dr Laura Jopson | 24/07/2023 |
| LIV20 | Liverpool University Hospitals NHS Foundation Trust | Aintree University Hospital | Dr Margaret Corrigan | 02/10/2023 |
| QMC03 | Nottingham University Hospitals NHS Foundation Trust | Queens Medical Centre | Peter Eddowes | 02/10/2023 |
| EDI33 | NHS Lothian | Royal Infirmary of Edinburgh | Mhairi Donnelly | 04/10/2023 |
| SOU14 | North Bristol NHS Trust | Southmead Hospital | Dr Ankur Srivastava | 06/11/2023 |
| UHB22 | University Hospitals Birmingham NHS Foundation Trust | Queen Elizabeth Hospital Birmingham | Dr Neil Rajoriya | 14/12/2023 |
| RFL12 | Royal Free London NHS Foundation Trust | Royal Free Hospital | Dr Rooshi Nathwani | 15/01/2024 |
| SHE10 | Sheffield Teaching Hospitals NHS Foundation Trust | Northern General Hospital | Dr Laura Harrison | 19/03/2024 |
| RLH34 | Barts Health NHS Trust | Royal London Hospital | Dr Vikram Sharma | 26/03/2024 |
| JCU40 | South Tees Hospitals NHS Foundation Trust | James Cook University Hospital | Dr Tim Hardy | 29/04/2024 |
| RSH 35 | Royal Surrey NHS Foundation Trust | Royal Surrey County Hospital, Guildford | Dr Marinos Pericleous | 08/05/2024 |
| UHD39 | County Durham & Darlington NHS Foundation Trust | University Hospital of North Durham (UHND) | Dr Francisco Porras-Perez | 05/06/2024 |
| DER36 | University Hospitals of Derby and Burton NHS Trust | Royal Derby Hospital | Dr Claire Grant | 18/06/2024 |
| RCH38 | Royal Cornwall Hospitals NHS Trust | Royal Cornwall Hospital | Dr Salma Mudawi | 01/07/2024 |
| NOR30 | Norfolk & Norwich University Hospitals NHS Foundation Trust | Norfolk & Norwich University Hospital | Dr Louisa Grant | 30/10/2024 |
| GAT41 | Gateshead Health NHS Foundation Trust | Queen Elizabeth Hospital | Dr Dina Mansour | 09/12/2024 |
| **SIV DONE** | | | | |
| WOL37 | The Royal Wolverhampton NHS Trust | New Cross Hospital | Dr Chris Corbett | - |
| **IN SET-UP** | | | | |
| OXF42 | Oxford University Hospitals NHS Foundation Trust | John Radcliffe | Francesca Saffioti | - |
| POR24 | Portsmouth Hospitals University NHS Trust | Queen Alexandra Hospital | Dr Richard Aspinall | - |
| WIG43 | Wrightington, Wigan and Leigh Teaching Hospitals NHS Foundation Trust | Royal Albert Edward Infirmary, Wigan | Dr Uche Nosegbe | - |
| **OPENED THEN SUBSEQUENTLY CLOSED** | | | | |
| NTY16 | Northumbria Healthcare NHS Foundation Trust | North Tyneside General Hospital | Avinash Aujayeb | 27/03/2023 |
