# Supplementary material for: Palliative long-term abdominal drains vs. large volume paracentesis for refractory ascites secondary to cirrhosis: protocol for a definitive randomised controlled trial (REDUCe2 study)
Source: Trials. 2025 Jun 4;26:193. doi: 10.1186/s13063-025-08873-z (PMC12139341; doi:10.1186/s13063-025-08873-z)
Supplement: Supplementary file 1 — Additional file 1. Appendices 1–7. [file 13063_2025_8873_MOESM1_ESM.zip › Appendix 2R1.pdf]

UNIVERSITY OF SUSSEX

brighton and sussex medical school

Palliative Long-term Abdominal Drains Versus REpeated Drainage in Untreatable Ascites Due To Advanced Cirrhosis: A Randomised Controlled Trial (REDUCe 2 Study)

Participant ID: __________

	Please initial box
I confirm that I have read and understand the information sheet version ____ for the above study-dated __/___/____. I have had the opportunity to consider the information, ask questions and have had these answered satisfactorily……………………………………	☐
I understand that my participation is voluntary and that I am free to withdraw at any time, without giving a reason and without my medical care or legal rights being affected………..	☐
I understand that relevant sections of my notes and data collected during the study may be looked at by individuals from the REDUCe 2 research team, University of Sussex, the Brighton and Sussex Clinical Trials Unit, regulatory authorities or local NHS trusts, where it is relevant to my taking part in this research. I give permission to these individuals to have access to my records…………………………………………………………………………	☐
I agree that my personal data included in this consent form will be reviewed outside my medical centre by responsible individuals designated by the Sponsor………………………..	☐
I consent to giving an additional four teaspoons of blood (20 ml) for research purposes. I understand that the blood will be stored in an anonymised manner. I understand that analysis of my blood samples may include genetic analysis (optional)………………………	Yes ☐ No ☐
I consent to having an interview with a researcher from SHORE-C and for my contact details to be passed onto them (optional)………………………………………………………..	Yes ☐ No ☐
I am happy for my caregiver/relative to be approached to be invited to take part in this research (optional)……………………………………………………………………………	Yes ☐ No ☐
I would like to receive a copy of the lay summary of the results (optional)………………….	Yes ☐ No ☐
I agree to my GP being informed of my participation in this study…………………………….	☐
I agree to take part in this study……………………………………………………………...	☐

For patients recruited in England and Wales only:

If I lose capacity during the study I agree for my caregiver or independent medical consultant to be contacted to advise if continuing in the study is in my best interests………	Yes ☐ No ☐

For patients recruited in Scotland only:

If I lose my capacity during the study I give my consent to continue in the study……………	Yes ☐ No ☐
If I lose my capacity during the study I give my consent for my already collected data to be used in the study…………………………………………………………………………….	Yes ☐ No ☐

____________________	____________	____________________
Name of Participant	Date	Signature

____________________	____________	____________________
Name of Researcher receiving consent	Date	Signature

UNIVERSITY OF SUSSEX

brighton and sussex medical school

<u>Consent Form</u>

Palliative Long-term Abdominal Drains Versus <u>RE</u>peated <u>D</u>rainage in <u>U</u>ntreatable Ascites Due To Advanced <u>C</u>irrhosis: A Randomised Controlled Trial (REDUCe 2 Study): Caregiver Questionnaire Study

Participant ID:

	**Please initial box**
I confirm that I have read and understand the information sheet version ____ for the above study-dated __/___/____. I have had the opportunity to consider the information, ask questions and have had these answered satisfactorily	☐
I understand that my participation is voluntary and that I am free to withdraw at any time, without giving a reason and without my medical care or legal rights being affected	☐
I agree to take part in the questionnaire study	☐
I agree for my contact details to be passed to the interview researcher	☐
I would like to receive a copy of the lay summary of the results	☐
I agree to take part in this study	☐

________________________	________________	________________________
Name of Patient	Date	Signature

________________________	________________	________________________
Name of Researcher receiving consent	Date	Signature

UNIVERSITY OF SUSSEX

brighton and sussex medical school

Palliative Long-term Abdominal Drains Versus REpeated Drainage in Untreatable Ascites Due To Advanced Cirrhosis: A Randomised Controlled Trial (REDUCe 2 Study)

PARTICIPANT INFORMATION SHEET AND CONSENT FORM – OPTIONAL ASCITIC FLUID SAMPLES

1. Introduction

We would like to invite you to take part in an optional aspect of the REDUCe2 trial available at your site. There are no additional risks and you will not have to undergo any additional procedures. Your participation in this is completely voluntary. Choosing not to participate will not affect your involvement in the main trial or the care you receive.

2. What Does Participation Involve?

Ascitic Fluid Collection: Half a cup (100 ml) of ascitic fluid will be collected at the start (baseline) and every four weeks until the end of the study follow-up period.

- **Long-term Drain Group:** fluid samples will be taken from the home drain. If any long-term abdominal drains are removed at any point during the course of your treatment, they may be collected and stored for further tests

- **LVP Group:** fluid samples will be taken from the hospital drain

3. What Will Happen to Any Samples I Give?

The ascitic fluid samples and any collected drains will be labelled with your study number, no other personal identifiers will be used. They will be stored at study sites and then sent to a Central Laboratory at the end of the study. They may be used in future research related to liver disease and its complications. Tests may include genetic markers. You will not be provided with any results of these tests as they are for research purposes only. Any surplus research samples will be destroyed at the end of the study in accordance with the hospital procedures.

CONSENT

Participant ID: __________	**Please <u>initial</u> box**
I confirm that I have read the information above and I have had the opportunity to consider the information, ask questions and have had these answered satisfactorily	☐
I agree to provide ascitic fluid sample for research and to the collection of any removed long term abdominal drains during or after trial follow-up. I understand that my participation is voluntary and that I am free to withdraw at any time without my care or rights being affected.	☐

______________________	________	______________________
Participant Name:	Date	Signature
______________________	________	______________________
Name of Researcher Receiving Consent	Date	Signature
