# Supplementary material for: Palliative long-term abdominal drains vs. large volume paracentesis for refractory ascites secondary to cirrhosis: protocol for a definitive randomised controlled trial (REDUCe2 study)
Source: Trials. 2025 Jun 4;26:193. doi: 10.1186/s13063-025-08873-z (PMC12139341; doi:10.1186/s13063-025-08873-z)
Supplement: Supplementary file 1 — Additional file 1. Appendices 1–7. [file 13063_2025_8873_MOESM1_ESM.zip › Appendix 3R1.docx]

**Appendix 3 –** Potential complications associated with the LTAD and recommended management protocol

| **Complication** | **Recommended management** | **Incidence observed in the REDUCe trial** | |
| --- | --- | --- | --- |
|  |  | **LTAD** | **LVP** |
| Leakage and cellulitis | Leakage usually self-limiting, if persists may need an extra suture. Continue ascites drainage via LTAD  Cellulitis usually results due to leakage and is again self-limiting. If persist may need a short course of antibiotics. Very rarely LTAD needs to be removed and can be re-sited | Leakage/cellulitis 41% | Leakage/cellulitis 11% |
| Suspected peritonitis | Do a diagnostic tap for cell count and culture from peritoneum as well as taking sample from LTAD. Treat as per usual peritonitis guidelines. Decision to remove LTAD must be made on a case-by- case basis after discussion with patient/caregiver  Routine sampling of ascitic fluid from LTAD and or routine blood tests in asymptomatic patients is not recommended. | 6% | 11% |
| Elevation in serum creatinine | Manage as clinically indicated | Serum creatinine (μmol/L) (median, IQR)  Baseline: 109 (79-141)  Week12: 104.5 (81-115.5) | Serum creatinine (μmol/L) (median, IQR)  Baseline: 113.5 (89-134)  Week 12: 127 (63-158) |
| LTAD blockage | Can be unblocked with flushing. If not admit to hospital and discuss need for replacement | 0% | NA |
| LTAD displacement | Admit to hospital if necessary and discuss need for replacement | 6% | NA |
| Bleeding | Usually self-limiting | 0% | 5% |
| Unable to manage ascites symptoms despite draining 1-2L three times a week from LTAD | Will need LVP in hospital - drain ascitic fluid via LTAD using adaptor with human albumin solution as per standard LVP protocols | 13% | NA |
