# Supplementary material for: Palliative long-term abdominal drains vs. large volume paracentesis for refractory ascites secondary to cirrhosis: protocol for a definitive randomised controlled trial (REDUCe2 study)
Source: Trials. 2025 Jun 4;26:193. doi: 10.1186/s13063-025-08873-z (PMC12139341; doi:10.1186/s13063-025-08873-z)
Supplement: Supplementary file 1 — Additional file 1. Appendices 1–7. [file 13063_2025_8873_MOESM1_ESM.zip › Appendix 4R1.docx]

**Appendix 4** – List of expected SARs

Expected SAR will include the following (only if they result in hospitalisation):

- LTAD or LVP leakage or blockage
- Cellulitis
- Pain at site of insertion not controlled by analgesia
- Bacterial peritonitis
- Sepsis which in the opinion of the PI is directly related to LTAD or LVP
- Death (only if in the opinion of the researchers directly related to the LTAD or LVP)
- > 50%increase in serum creatinine from baseline
- Electrolyte imbalances with or without increase in serum creatinine
- Bleeding if directly related to LTAD or LVP
- Bowel perforation if directly related to LTAD and LVP
- Failed drainage and or drain displacement
