# Supplementary material for: Palliative long-term abdominal drains vs. large volume paracentesis for refractory ascites secondary to cirrhosis: protocol for a definitive randomised controlled trial (REDUCe2 study)
Source: Trials. 2025 Jun 4;26:193. doi: 10.1186/s13063-025-08873-z (PMC12139341; doi:10.1186/s13063-025-08873-z)
Supplement: Supplementary file 1 — Additional file 1. Appendices 1–7. [file 13063_2025_8873_MOESM1_ESM.zip › Appendix 6R1.docx]

**Appendix 7** – List of protocol amendments

| **AMENDMENT NUMBER** | **TYPE OF AMENDMENT (Substantial or Non-Substantial)** | **DATE OF HRA APPROVAL** | **AMENDMENT SUMMARY (including listing new document versions)** |
| --- | --- | --- | --- |
| 01 | Non-Substantial | 26^th^ August 2022 | Addition of wording about what happens to a participant’s blood samples in Section 14 of the Participant PIS (V3.0 25^th^ August 2022), addition of 6 sites |
| 02 | Non-Substantial | 03^rd^ November 2022 | Addition of sites. Clarification of the protocol/ schedule of events and the addition of a questionnaire to collect demographic information about caregivers. Protocol V3.0 24Oct02022, Some PIs have been updated. |
| 03 | Non-Substantial | 20^th^ December 2022 | Changes to Child Pugh Score and addition of Community Centres being used for fortnightly research visits Protocol V4.0 23Nov2022 |
| 04 | Non- substantial | 16^th^ January 2023 | Added 2 sites, replaced PI at one site as a temporary measure to cover maternity leave |
| 05 | Non- substantial | 27^th^ February 2023 | Update of protocol to allow use of 2 manufacturers of the LTADs – Rocket Medical and Beckton Dickinson |
| 06 | Non- substantial | 5^th^ June 2023 | Update of PIS & Patient Consent form, changes to Modified AHCR questionnaire, translation of patient PIS and patient consent into Urdu and translation of patient PIS, patient consent, caregiver PIS, caregiver consent, consultee cover letter and Consultee Record of Consultation in to Welsh. |
| 07 | Non-substantial | 23^rd^ August 2023 | Clarification of Inclusion Criteria, clarification of threshold for an expected SAR with respect to serum creatinine levels to reflect current definition of AKI in cirrhosis, amendment to typos in the pilot study details, update of the Sponsor contact and BSCTU director, update of the status of the study statisticians. |
| 08 | Non-substantial | 13^th^ November 2023 | Added 3 new sites, replaced PI at one site |
| 09 | Non-substantial | 30^th^ January 2024 | Translation of patient PIS and patient consent into Bengali, change of PIs at 6 sites, amendment of wording in Protocol V7.0 18^th^ Dec 2023 - post-LTAD insertion, change of wording to include drainage bottles and addition of guidance for LTAD suture removal. |
| 10 | Non-substantial | 9^th^ April 2024 | Amendment of wording in Protocol V8.0 14^th^ March 2024 to clarify that electrolyte imbalances are also seen in patients with advanced cirrhosis and that in cases of a blocked LTAD only to admit to hospital if the drain cannot be unblocked by flushing in the community.  Also, the addition of an information sheet, consent form and pre- and post-workshop questionnaires in the workshops for site staff being run by SHORE-C.  The addition of a site. |
| 11 | Non-substantial | 6th August 2024 | Replaced PI at two sites and translation of patient PIS and patient consent into Turkish and Punjabi |
| 12 | Substantial | 15th October 2024 | Amendment of wording in Protocol V9.0 to remove any reference to ‘draining to dryness’ to avoid it being misinterpreted by teams. Changes to the protocol to add the collection of optional research ascitic fluid at specific sites (at baseline and 4 weekly). This is accompanied by a standalone participant information sheet and consent form for these optional tests |
