# Supplementary material for: Palliative long-term abdominal drains vs. large volume paracentesis for refractory ascites secondary to cirrhosis: protocol for a definitive randomised controlled trial (REDUCe2 study)
Source: Trials. 2025 Jun 4;26:193. doi: 10.1186/s13063-025-08873-z (PMC12139341; doi:10.1186/s13063-025-08873-z)
Supplement: Supplementary file 1 — Additional file 1. Appendices 1–7. [file 13063_2025_8873_MOESM1_ESM.zip › Appendix 7R1.docx]

**Appendix 8** – STOP/GO criteria of internal pilot

(sections in **bold** specifying what was met in the pilot)

|  | **RED** | **AMBER** | **GREEN** * |
| --- | --- | --- | --- |
| Pilot recruitment/total study recruitment target by end of study month 21 | <10% | 10-20% | **>20%** |
| Mean recruitment rate/site/month (based on data to end of study month 21) | <0.2 | 0.2-0.3 | **>0.3** |
| Number of sites opened by end of study month 18 | <19 | **20-23** | ≥24 |
| Total number of patients recruited by study month 18 | <37 | 37-47 | **>47** |
| Proportion of those recruited by study month 18 retained to end of follow up* | <50% | 50%-60% | **>60%** |
| Percentage of LTADs removed due to peritonitis by end of study month 21* | >15% | 10%-15% | **<10%** |
| Proportion completing SFLDQoL questionnaire (except questions on sexual function) in those retained at time point, by end of month 18*  Baseline  Week 4  Week 8  Week 12 | <91%  <85%  <80%  <75% | 91%-95%  **85%-90%**  80%-85%  75%-80% | **>95%**  >90%  **>85%**  **>80%** |
| Checklist for LTAD insertion and post procedure monitoring % completed | <75% | 75%-85% | **>85%** |
